# Acute small bowel perforation after wireless capsule endoscopy in a patient with Crohn’s disease: a case report

**DOI:** 10.4076/1757-1626-2-7607

**Published:** 2009-07-31

**Authors:** Dhavan A Parikh, Janak A Parikh, Gregory C Albers, Charles F Chandler

**Affiliations:** 1 Department of Internal Medicine, University of California Davis Health System4860 Y Street, Sacramento, California 95817USA; 2 Department of General Surgery, University of California Los Angeles Medical Center200 UCLA Medical Plaza, Los Angeles, CA 90095USA; 3 Department of Gastroenterology, University of California Irvine Medical Center101 The City Drive, Orange CA 92868USA

## Abstract

**Introduction:**

Wireless capsule endoscopy is an important tool for minimally invasive evaluation of the small bowel, allowing improved diagnostic yield with low complication rates relative to traditional modalities. Recently however, reports on small bowel perforation after wireless capsule endoscopy have surfaced. Here we present the first case of acute small bowel perforation in a middle-aged male in the United States.

**Case presentation:**

A 58-year-old male with a presumed quiescent history of Crohn’s Disease presented to the Emergency Department in a septic state 48 hours after a wireless capsule endoscopy procedure complaining of abdominal pain, distension, and frequent emesis. A computed tomography scan of the abdomen was suggestive of small bowel perforation and ischemic enteritis. The patient was adequately resuscitated and taken to the operating room for an ileocecectomy and extensive resection of the small bowel. Pathology of the resected specimen revealed an ileal stricture and associated necrotizing ileitis, and a perforation just proximal to the stricture.

**Conclusion:**

Wireless capsule endoscopy remains the preferred endoscopic imaging method of the small bowel. This case illustrates the importance of appropriate patient selection and evaluation of functional patency of the small bowel prior to wireless capsule endoscopy, especially with the growing role of this procedure in the evaluation of inflammatory bowel disease.

## Introduction

Since its conception in 2000, wireless capsule endoscopy (WCE) has become an established tool for minimally invasive evaluation of the small bowel (SB). Today WCE is most commonly utilized for evaluation of obscure GI bleeding unidentified by traditional endoscopic techniques [1]. In recent years however, an advancing body of literature advocating the use of WCE in the diagnosis and management of inflammatory bowel disease (IBD) has emerged. Studies have reported on the utility of WCE for the initial diagnosis and staging of Crohn’s Disease (CD), evaluation of post-operative CD, assessment of treatment efficacy, and investigation of patients with indeterminate colitits [2-4].

While WCE is a generally safe and well tolerated procedure in patients with IBD, it does carry the potential for serious complications. Capsule retention is a well recognized complication occurring at a higher frequency in patients with known or suspected CD [1]. Capsule impaction leading to clinical SB obstruction has also been reported in the literature as a serious complication requiring immediate surgical intervention [5]. Three cases of SB perforation after WCE have recently been reported in the elderly, two involving patients with CD [6-8]. Recipi et al. describe an acute SB perforation in an 82 year old male with suspected CD, while Um et al. report a SB perforation in a 75 year old female with well established active CD 17 days post-procedure. Here we report the first case of acute SB perforation after WCE in the United States in a patient with a presumed quiescent history of CD.

## Case presentation

A 58-year-old Caucasian male with a reported 30 year history of CD presented to his gastroenterologist with intermittent diarrhea controlled by bismuth subsalicylate (Kaopectate). He denied any recent flares or pharmacologic management. A colonoscopy was negative for classical findings of CD and the patient next underwent WCE to document possible disease in the SB. On the evening of the procedure the patient reported poorly localized abdominal pain and copious non-bilious/non-bloody emesis. Over the next 48 hours he experienced progressive abdominal distention and presented to the Emergency Department. He denied the chronic use of NSAIDs or a history of prior abdominal operations.

**Figure 1.**
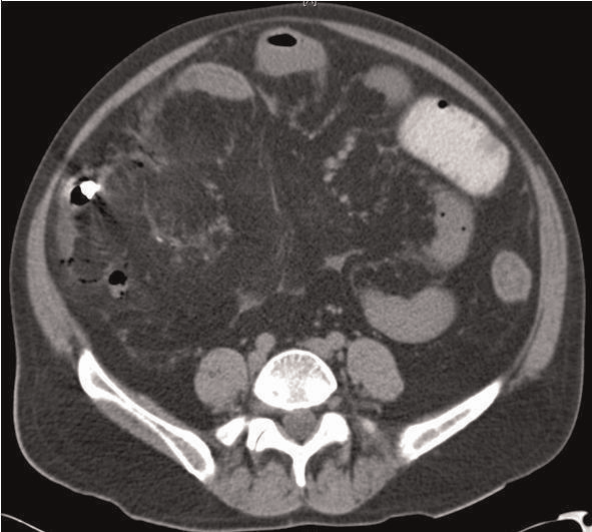
Small bowel perforation caused by capsule endoscope. Contrast-enhanced CT scan of the abdomen taken on admission showing loculated free air, fat stranding and a capsule endoscope near the terminal ileum (white arrow).

On admission the patient was febrile, tachycardic and tachypneic. He also presented with severe hypotension requiring vasopressors. His physical exam was positive for icteric sclera, a distended abdomen with increased bowel sounds, and marked tenderness in the right lower quadrant. He had a WBC of 13.9 × 10^9^/L (4.5-11.0 × 10^9^/L) and platelet count of 79 × 10^9^/L (150-450 × 10^9^/L). His creatinine was 203.3 umol/L (58-111 umol/L) and total bilirubin was 171 umol/L (3-22 umol/L). A CT scan of the abdomen/pelvis revealed a capsule endoscope located near the terminal ileum along with diffusely thickened bowel wall, fat stranding in the right lower quadrant, hemorrhagic ascities and loculated free air (Figure 1), reported as ischemic enteritis and SB perforation.

After adequate resuscitation the patient was taken to the operating room where an ileocecectomy was performed, including removal of 160 cm of SB. The capsule endoscope found just proximal to the stricture. Given the patient’s hemodynamic instability the abdomen was initially closed with a 2 mm GORE-TEX (WL Gore & Associates Inc., Flagstaff, AZ) patch and loose suture. He required 12 subsequent operations for various complications and was discharged after 1 month with an ileostomy and colostomy. Pathology of the resected specimen showed an ileal stricture 15 cm proximal to the ileocecal valve, with associated necrotizing ileitis. A perforation was noted just proximal to the stricture where the capsule endoscope was found.

## Discussion

WCE is a safe and well tolerated procedure with few complications. Over 400,000 capsules have been deployed worldwide since 2000 with rare complications and no reported deaths [9]. To date, the most common complication is retention of the capsule, ranging from 0% to 13% of all cases, with larger single institution studies reporting a rate of up to 2.5% [1,9]. Patients at high risk for capsule retention include those with radiation enteritis, known CD, SB tumors, NSAID enteritis, SB diaphragm disease, prior SB obstruction, and those with prior SB resection and primary anastamosis [1]. Although a time limit has not been set for intervention after documented capsule retention, surgical and enteroscopic retrieval have proven beneficial for removal of the capsule endoscope and correction of the underlying pathology in symptomatic patients [10]. In asymptomatic patients surgical retrieval may be indicated if the identified pathology can be treated operatively.

Despite concerns over capsule retention and possible obstruction, the widespread use of WCE for evaluation of CD is justified by improvements in diagnostic yield relative to traditional modalities, with low procedure risk. Overall diagnostic yield has commonly been reported between 15% and 44%, and a recent case series has shown a diagnostic yield of 86% leading to a change in management in 64% of cases [11,12].

In the present case, capsule impaction secondary to a stricture in the terminal ileum was likely the inciting mechanism leading to acute SB obstruction and subsequent perforation. The use of more rigorous pre-procedure evaluation in patients with suspected or known CD should therefore be pursued. Specifically, additional imaging in the form of barium follow-through, and CT/MR enterography may help characterize SB disease, although the presence or absence of a stricture does not necessarily preclude the possibility of SB obstruction. In this regard, the patency capsule has shown promise for the evaluation of patients with known strictures or other SB disease leading to obstruction [13]. Spada et al. showed a 100% video capsule passage rate in 10 patients with known strictures, screened from an original pool of 34 patients by the Agile Patency Capsule (Given Imaging, Yoqneam, Israel) [14]. Although the patency capsule itself has also been shown to result in symptomatic SB obstruction in a few cases [15], appropriate utilization and interpretation with this system is valuable in evaluating the functional patency of WCE candidates.

The advent of WCE has revolutionized imaging of the SB, improving diagnostic yield at relatively low complication rates. Our report, in combination with the Repici and Um reports, suggests that more serious complications than previously anticipated exist for this technique and may not be limited to the elderly. Furthermore, SB perforation should be recognized as a possible complication in patients with well established, suspected, or even a presumed quiescent history of CD. Capsule endoscopists must exercise extended caution in patient selection, especially as utilization of WCE increases in the management of IBD.

## Abbreviations

CD: Crohn’s disease
CT: computed tomography
IBD: inflammatory bowel disease
MR: magnetic resonance
NSAIDs: non-steroidal anti-inflammatory drugs
SB: small bowel
WCE: wireless capsule endoscopy
